# An atypical phenotype of Reis-Bücklers corneal dystrophy caused by the G623D mutation in *TGFBI*

**Published:** 2008-07-11

**Authors:** Dandan Li, Yanhua Qi, Li Wang, Hui Lin, Nan Zhou, Liming Zhao

**Affiliations:** Department of Ophthalmology, Harbin Medical University the 2nd Affiliated Hospital, Harbin, China

## Abstract

**Purpose:**

To characterize the molecular defects in the *TGFBI* gene in a Chinese family with an unusual phenotype of Reis-Bücklers corneal dystrophy (RBCD).

**Methods:**

Three generations of the family with RBCD were enrolled in the present study. In addition to ophthalmologic examinations, polymerase chain reaction (PCR) amplification and nucleotide sequencing of exons of *TGFBI* were performed. Exon 14 was also sequenced in 100 healthy controls unrelated to the family for comparison.

**Results:**

The clinical features of the disease were characterized by geographic opacities in the anterior to mid stroma of the cornea. Molecular genetic analysis revealed a heterozygous point mutation at exon 14 (c.1915 G>A) in all affected members of the family. The unusual opacities involving the anterior to mid-cornea stroma were different from the phenotypic features of families previously reported to have the same genetic change.

**Conclusions:**

We speculate that this disorder is a variant of RBCD. This finding may expand our knowledge about RBCD and facilitate diagnosis of corneal dystrophies associated with atypical clinical features.

## Introduction

Corneal dystrophy is a group of hereditary disorders characterized by bilateral abnormal deposition in different layers of the cornea which may impair corneal transparency and refraction [1]. Most of the corneal dystrophies are inherited as autosomal dominant traits with extensive intrafamily and interfamily variations in clinical phenotypes [2]. The transforming growth factor beta induced gene (*TGFBI*, OMIM 601692), located on chromosome 5q31, has been linked to many types of corneal dystrophy including lattice corneal dystrophy of types I, IIIA, I/IIIA, IIIB, and IV, granular corneal dystrophy type I, Avellino corneal dystrophy, Thiel-Behnke dystrophy, and Reis-Bücklers dystrophy [3-8]. Particular mutations in *TGFBI* are frequently linked to special corneal dystrophy, but a genotype-phenotype correlation is not always certain [9-12]. Atypical and variable phenotypes have made it difficult to accurately diagnose and classify the corneal dystrophies particularly for patients with equivocal or ambiguous findings in the clinic.

Reis-Bücklers corneal dystrophy (RBCD, OMIM 608470) is a dominantly inherited dystrophy that primarily affects Bowman’s layer and is characterized by frequent recurrence, painful corneal erosions, superficial corneal opacities, and significant visual impairment [13,14]. To date, four different mutations in *TGFBI* have been found to be associated with most cases of RBCD [15-17].

**Figure 1:**
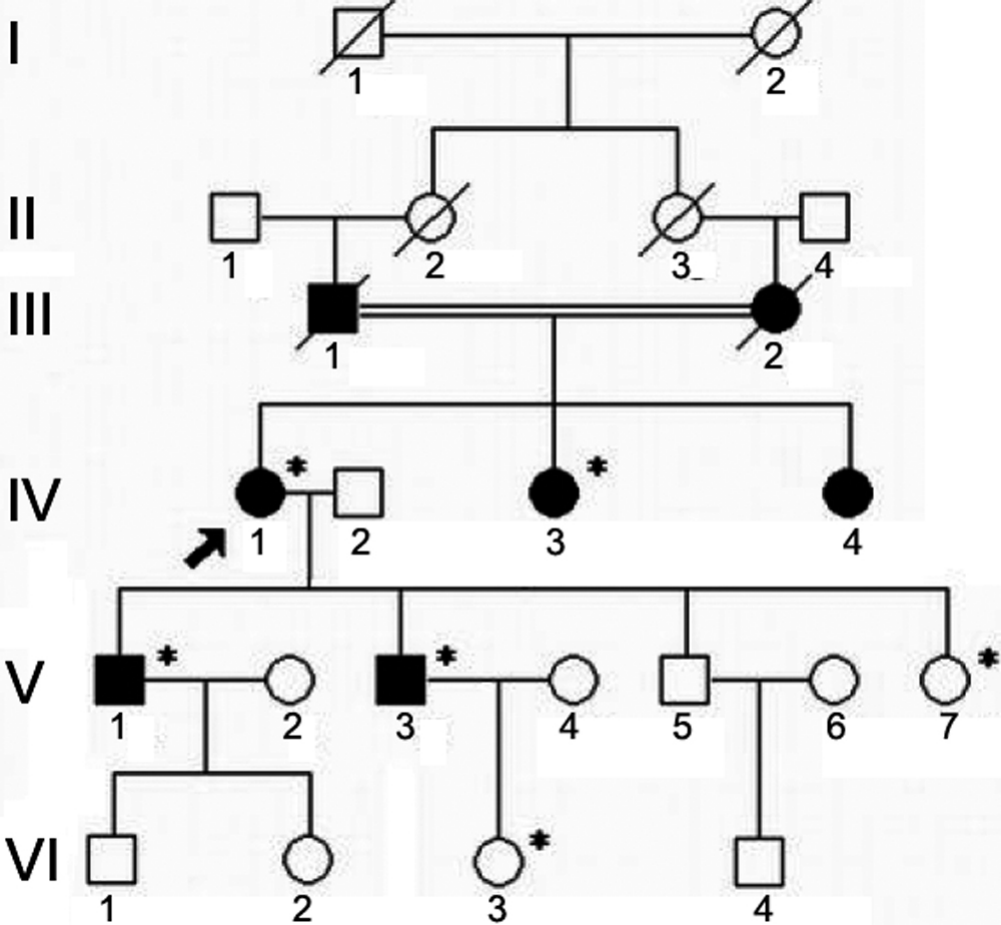
Pedigree of the proband’s family. Autosomal dominant transmission of the disease is evident. An asterisk indicates the subject underwent clinical and molecular analyses. Black/closed symbols represent the affected members. The arrow signals the proband.

RBCD is relatively rare in China, and limited studies have been reported [18,19]. Thus far, the R124L mutation in *TGFBI* is the only one found to be associated with this type of corneal dystrophy in Chinese patients. In this study, we describe a Chinese consanguineous family with an atypical RBCD possibly caused by the p.G623D mutation. The unusual opacities involving the anterior to mid-cornea stroma are different from the phenotype previously reported for this mutation. We propose that this particular disorder may be a variant of RBCD. This finding expands the genotypic-phenotypic spectrum of RBCD and may facilitate diagnosis of this disease in future.

**Table 1:** Primers used for *TGFBI* amplification.

**Exon**	**Sequences (5′-3′)**	**Annealing Temp (°C)**	**Reference**
1	F: GCGCTCTCACTTCCCTGGAG	53	[8]
	R: GACTACCTGACCTTCCGCAG		
2	F: GGTGGACGTGCTGATCATCT	55	[8]
	R: AGCCAGCGTGCATACAGCTT		
3	F: ACCTGTGAGGAACAGTGAAG	55	[22]
	R: GCCTTTTATGTGGGTACTCC		
4	F: CCCCAGAGGCCATCCCTC CT	59	[3]
	R: CCGGGCAGACGGAGGTCATC		
5	F: TAAACACAGAGTCTGCAGCC	55	[21]
	R: TTCATTATGCACCAAGGGCC		
6	F: TGTGTTGACTGCTCATCCTT	50	[21]
	R: CATTCAGGGGAACCTGCTCT		
7	F: TTCAGGGAGCACTCCATCTT	55	[21]
	R: ATCTAGCGCACAAATGAGG		
8	F: CTTGACCTGAGTCTGTTTGG	53	[8]
	R: GAAGTCGCCCAAAGATCTCT		
9	F: ACTTTTGAACCCACTTTCTC	55	[21]
	R: CAATCTAACAGGGATGCCTT		
10	F: TCTGGACCTAACCATCACCC	55	[21]
	R: CAGGAGCATGATTTAGGACC		
11	F: CTCGTGGAAGTATAACCAGT	55	[3]
	R: TGGGCAGAAGCTCCACCCGG		
12	F: CATTCCAGTGGCCTGGACTCTACTATC	58	[3]
	R: GGGGCCCTGAGGGATCACTACTT		
13	F: GGGATTAACTCTATCTCCTT	50	[8]
	R: TGTGTATAATTCCATCCTGG		
14	F: CTGTTCAGTAAACACTTGCT	55	[3]
	R: CTCTCCACCAACTGCCACAT		
15	F: CACTCTGGTCAAACCTGCCT	55	[21]
	R: AGGCTAGGCGCAAACCTAGC		
16	F: CAGTTGCAGGTATAACTTTC	58	[21]
	R: TAAACAGGTGTGCAATGACT		
17	F: GGGAGATCTGCACCTATTTG	55	[8]
	R: TGGTGCATTCCTCCTGTAGT		

Summary of the primers and annealing temperatures used for the amplification of the 17 exons of *TGFBI*.

## Methods

### Patients and controls

We studied a three-generation Chinese family with RBCD from the Heilongjiang province in northeastern China (Figure 1). Four patients, two non-carrier relatives, and 100 healthy unrelated normal controls were recruited in this study. The study was approved by a local institutional review board, and informed consent was obtained from each of the participants. All subjects underwent ophthalmologic evaluation including best corrected visual acuity, slit lamp, and fundus examination.

### Genetic analysis

Blood samples were drawn by venipuncture, and genomic DNA was extracted using the TIANamp Blood DNA Kit (Tiangen Biltech Co. Ltd, Beijing, China). All exons of *TGFBI* were amplified by polymerase chain reaction (PCR) using the primers described previously [8,20-22]. The sequences of the primers are listed in Table 1. The PCR amplification reaction mixture (50 μl) contained 10X PCR buffer, 10–200 ng of genomic DNA, 0.2 mM of each dNTP, 1 unit of Taq polymerase, and 1 μl of 1 mM forward and reverse primers. The thermocycling procedure included an initial denaturation step at 95 °C for 5 min followed by 35 cycles of denaturation, annealing, and extension. The primer annealing temperatures are shown in Table1, and the terminal extension step was accomplished at 72 °C for 10 min. The PCR products were purified with a TIANgel Midi Purification Kit (Tiangen Biltech Co. Ltd, Beijing, China) and sequenced with an ABI BigDye Terminator Cycle Sequencing kit v3.1, (ABI Applied Biosystems, Foster City, CA).

## Results

### Clinical findings

**Figure 2:**
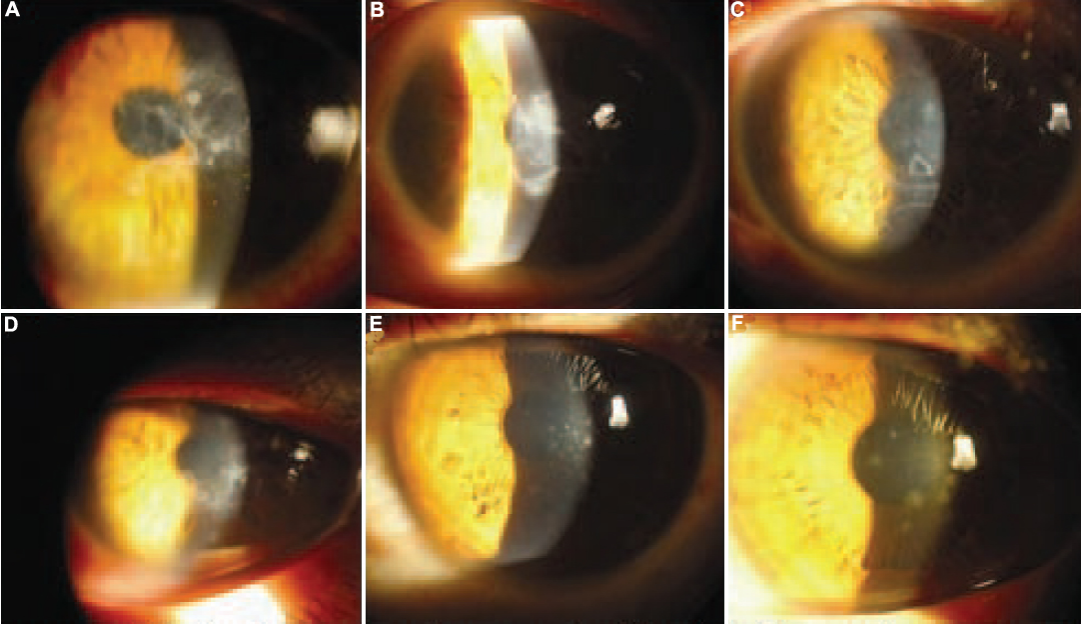
Clinical photography of corneas in affected family members. **A** and **B**: Slit lamp photographs of the proband show multiple opacities in the subepithelial and anterior stromal regions in the central cornea of the left eye (**A)** and geographical opacity involving the anterior and mid stroma in the right eye **(B)**. **C** and **D**: Corneal images of patient IV:3 revealed geometric and round opacities involving the anterior stroma and subepithelial layers in both eyes. **E** and **F**: Corneal images of V:1 and V:3 are shown, respectively. Dot epithelial and subepithelial opacities were noted.

The proband (Patient IV: 1, Figure 1) was a 56-year-old woman who was the offspring of a consanguineous marriage. She had experienced defective vision along with corneal erosion since the age of 32. Corneal examination revealed multiple opacities in subepithelial and anterior stroma in the central cornea of the left eye (Figure 2A). In the right eye, a gray-white geographic opacity involving the anterior and mid stroma of the cornea with clear borders was noted (Figure 2B). Patient IV:3, the 53-year-old sister of the proband, was diagnosed with the disease at age 30. Anterior segment evaluation revealed geometric opacities mixed with round haze in the anterior stroma and subepithelial layers of the central cornea in both eyes (Figures 2C,D). Two other subjects evaluated were from the fifth generation (V:1 and V:3; 33 and 31 years old, respectively) and had no clinical symptoms but manifested diffuse, small dot epithelial and subepithelial opacities bilaterally in the central cornea (Figures 2E,F). V:7 and VI:3 did not show any clinical sign of corneal dystrophy.

### *TGFBI* mutation analysis

**Figure 3:**
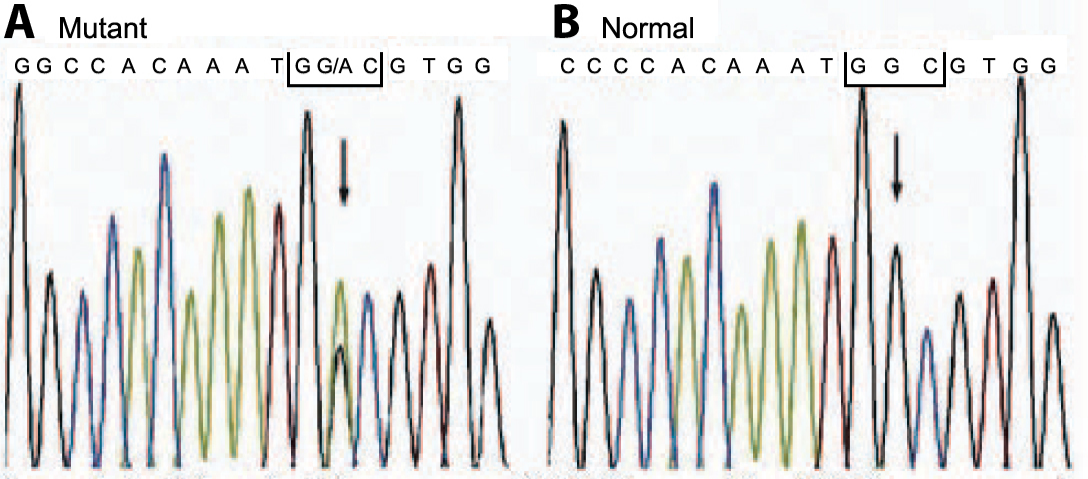
Partial nucleotide sequence of *TGFBI* exon 14. **A**: The sequence in an affected subject shows a heterozygous G>A transversion (indicated by the arrow). The nucleotide substitution at codon 623 resulted in a change from glycine (GGC) to aspartic acid (GAC). **B**: Unaffected family members and the control subjects lacked this nucleotide change.

After direct sequencing of exons of *TGFBI*, a single heterozygous G>A mutation at nucleotide position 1915 in exon 14 of *TGFBI* (codon 623) was detected (Figure 3). This resulted in a glycine to aspartic acid substitution at the protein level. None of the healthy family members or any of the 100 control subjects was positive for this mutation.

## Discussion

To date, four distinct autosomal dominant Bowman’s layer/stromal corneal dystrophies have been linked to various mutations in *TGFBI*. A phenotype-genotype correlation between specific types of corneal dystrophies and particular *TGFBI* mutations has been established among these patients [23]. However, corneal dystrophies also sometimes display a wide range of clinical manifestations even when the same single point mutation is involved [3,9-12]. The variations in phenotype and penetrance can lead to difficulties in classification, diagnosis, and pathological study of these diseases. In this study, we demonstrated an unusual form of RBCD associated with the p.G623D mutation in *TGFBI*.

Three families with the p.G623D mutation were previously described in the literature [3,8,24]. In 2001, Afshari et al. [8] first described a family with RBCD caused by the p.G623D mutation. The patient had a history of recurrent painful corneal erosion since childhood and showed geographic subepithelial corneal opacities. The following year, Munier et al. [3] reported on another family carrying the same mutation. However, based on the late onset of the disease and the presence of tiny linear deposits in the anterior stroma, this type of corneal dystrophy has been reclassified as lattice corneal dystrophy I/IIIA. An additional family with the p.G623D mutation was described by Aldave et al. in 2004 [24]. The family had a unique corneal dystrophy involving Bowman’s layer and the corneal stroma. Affected members had subepithelial haze mixed with lattice line-like opacities in the anterior stroma.

The pedigree reported herein was from a consanguineous marriage. The proband had a gray-white geographic opacity in the anterior to mid stroma of her right eye. In addition, geometric and round opacities involving the anterior stroma and subepithelial layers were also found in both the proband and her sister. The clinical features including recurrent erosion and gradually developing opacities of Bowman’s layer were identical to the characteristics of RBCD [13,14] and to those found in the families described previously by Afshari et al. [8] and Aldave et al. [24]. However, no lattice lines were noted in the proband or other affected members, and the deposits were located in the mid stroma of the cornea, which was different from the phenotypes previously reported for this mutation. Thus, we propose that this particular disorder may be a variant of RBCD.

**Figure 4:**
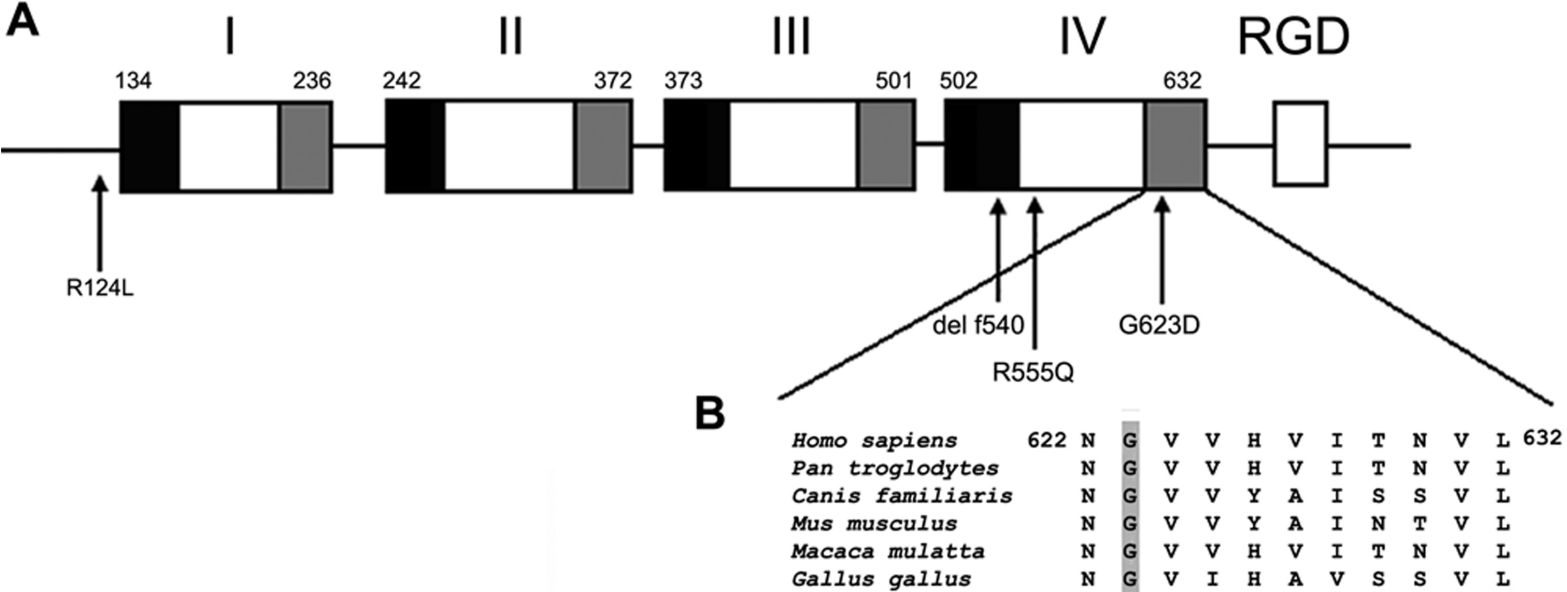
Schematic diagram of TGFBI. **A**: A diagram of recombinant ßIG-H3 proteins is illustrated. Black and gray boxes indicate the highly conserved sequences of each repeated domain. The RGD motif is shown as an open box. Mutations of *TGFBI* associated with RBCD occur at amino acids 124, 555, 540, and 623 are indicated by arrows. **B**: The alignment of *TGFBI* sequences in diverse species is shown. The glycine is conserved in TGFBI proteins from several species.

The protein encoded by *TGFBI* is βig-h3 (TGFBIp, Keratoepithelin), which consists of four tandem repeat domains of 140 residues and a COOH-terminal Arg-Gly-Asp (RGD) motif. The protein has a remarkable sequence similarity to the *Drosophila melanogaster* axonal guidance molecule, fasciclin 1 (FAS1) [25] (Figure 4). The four FAS1 domains in human TGFBIp correspond to amino acids 134–236 (FAS1–1), 242–372 (FAS1–2), 373–501 (FAS1–3), and 502–632 (FAS1–4). The p.G623D mutation is located in the fourth internal domain of TGFBIp. The glycine residue is highly conserved in several species including *Pan troglodytes*, *Canis familiaris*, *Mus musculus*, *Macaca mulatta*, and *Gallus gallus* (Figure 4), suggesting that a mutation in this region may have a severe consequence in the structure and/or the function of the protein. Moreover, in 2003, Clout NJ et al. [26] made a model of the FAS1 domain 4 of βig-h and found that Gly623 is located in β7. The mutation of glycine to aspartic acid is incompatible with correct folding of FAS1, and the mutated G623D βig-h3 is unlikely to be secreted.

Several factors such as advanced stage disease, age, environmental factors, and modifier genes contribute to the intrafamily and interfamily variability in phenotypic expression. Thus, a precise diagnosis of corneal disorders cannot rely solely on the clinical and histopathological features. In this study, we provide clinical and molecular evidence supporting the occurrence of a distinct RBCD phenotype attributable to the *TGFBI* p.G623D mutation. These data enlarge the range of phenotypic variability of RBCD associated with mutations in *TGFBI* and expand our knowledge of RBCD in Chinese patients. These findings may be helpful for improving the diagnosis of corneal dystrophies associated with atypical clinical features.
